# The pharmacokinetics and Concentration-QTc analysis of a new etomidate analog ET-26-HCl: a phase I trial in healthy Chinese subjects

**DOI:** 10.3389/fphar.2025.1534717

**Published:** 2025-04-17

**Authors:** Kun He, Wenyu Zhang, Xiangran Kong, Lize Li, Lei Diao, Qing Wen, Guohai Su, Xiaoran Yang, Hongyan Zhao

**Affiliations:** ^1^ Department of Clinical Research Center, Central Hospital Affiliated to Shandong First Medical University, Jinan, China; ^2^ Department of Cardiovascular Medicine Central Hospital Affiliated to Shandong First Medical University, Jinan, China; ^3^ Avanc Pharmaceutical Co., Ltd., Jinzhou, China; ^4^ Shanghai Fosun Pharmaceutical Development Co. Ltd., Shanghai, China

**Keywords:** C-QTc modeling, pharmacokinetics, methoxyetomidate hydrochloride, QTc effect, etomidate acid

## Abstract

Methoxetamine hydrochloride (ET-26-HCl) is a novel short-acting intravenous general anesthetic that retains the advantages of etomidate while minimizing its impact on adrenal cortical function. A single-center, randomized, open-label, placebo-controlled clinical trial was conducted using concentration-QTc (C-QTc) model analysis to evaluate the pharmacokinetics, clinical sedative effect, safety, and potential risk of QT interval prolongation of ET-26-HCl at doses of 0.8 mg/kg (the clinical dosage) and 2.8 mg/kg. In the 0.8 mg/kg group, the mean peak concentration (*C*_max_) of ET-26 was 1,510 ng/mL with upper limits of the 90% confidence interval (CI) for QTcF interval corrected by baseline and placebo (ΔΔQTcF) falling within an acceptable range, not exceeding ±10 ms (−1.543 ms to +2.788 ms). The 2.8 mg/kg group exhibited a higher *C*_max_ value for ET-26, along with corresponding mean ΔΔQTcF values that remained below the ±10 ms threshold limit. Based on the established C-QTc model analysis, it is predicted that the upper limit of 90% CI for the mean ΔΔQTcF corresponding to ET-26 at twice the *C*_max_ of 0.8 mg/kg is ≤ ±10 ms. The study findings in conjunction with the C-QTc model demonstrated the rapid onset and recovery properties of ET-26. Furthermore, increased exposure and dose-dependent sedative/hypnotic effects were observed, with no risk of QT prolongation for this investigational drug, thereby ensuring patient safety and minimizing potential risks in its clinical application.

**Clinical Trials Registration Number:**

ClinicalTrials.gov CTR20233230.

## 1 Introduction

The incidence of cardiac adverse events (AEs) caused by non-antiarrhythmic drugs in clinical trials is low; however, the risk of these events is increased with a prolonged QT interval on electrocardiography (ECG), which can potentially lead to torsades de pointes (Gupta et al., 2007). The International Council for Harmonization of Technical Requirements for Pharmaceuticals for Human Use (ICH) released the E14 guidelines in 2005, recommending thorough QT studies (TQT) to assess the impact on the QT interval and mitigate the risk of malignant arrhythmias after market approval for new drugs with systemic bioavailability (ICH, 2005a; ICH, 2005b). Subsequently, both the ICH and the Food and Drug Administration (FDA) published a series of guidelines and scientific white papers acknowledging Concentration-QTc (C-QTc) modeling as an alternative approach to TQT studies (ICH, 2015; ICH, 2022; Garnett et al., 2017; Garnett et al., 2018). This approach guides the early evaluation of cardiac safety because of its potential for cost reduction, shorter duration, and decreased false-positive rates (Bouvy et al., 2011; Cavero et al., 2016). Consequently, an increasing number of investigational drugs have been examined using C-QTc modeling analysis.

Methoxyetomidate hydrochloride (ET-26-HCl) is a newly developed, short-acting intravenous general anesthetic belonging to the imidazole class. Its active ingredient, methoxyetomidate (ET-26), has a structure similar to that of etomidate and retains the advantages of respiratory and cardiovascular stability, as well as a wider safety range; however, this weakens the inhibitory effect on adrenal cortex function (Zhang et al., 2019; Zhang Y. et al., 2020; Zhang Y. J. et al., 2020). ET-26-HCl is reported to maintain the superior myocardial performance of etomidate (Liu et al., 2018). Additionally, ET-26 exhibits high lipid solubility, allowing it to cross the blood–brain barrier and enhance the function of GABAa receptors containing β2 or β3 subunits (Yu et al., 2020). This induction leads to inhibitory excitatory potentials, opening chloride ion channels and strengthening the inhibitory effect of GABA neurotransmitters, ultimately producing anesthetic effects (Jiang et al., 2017; Rudolph et al., 2017).

It is widely recognized that most compounds that prolong the QT interval inhibit the cardiac rapid delayed rectifier potassium current (IKr), which is encoded by the human ether-à-gogo gene (*hERG*) and plays a crucial role in defining ventricular repolarization, closely associated with potential arrhythmias (Sanguinetti et al., 1995; Trudeau et al., 1995). *In vitro* experiments have demonstrated that ET-26-HCl has an IC50 value greater than 102.11 µM for hERG potassium channels (Liu et al., 2018), which is significantly higher than the drug concentration required to produce anesthesia effects and is unlikely to cause significant QT interval prolongation. In addition, a study involving beagle dogs found no toxicologically significant prolongation of QTc intervals compared with pre-administration values after a single intravenous injection at different doses (8, 12, and 16 mg/kg) of ET-26-HCl (Zhang et al., 2019). A clinical trial was conducted in healthy subjects to evaluate the impact of ET-26-HCl on the QT interval in humans, despite previous preclinical investigations not showing evidence of QT interval prolongation. This study utilized a C-QTc effect model to predict the influence of ET-26 on the QT interval because of its non-antiarrhythmic drug properties. Additionally, this study aimed to investigate the pharmacokinetic (PK) properties of ET-26-HCl and assess its cardiac safety profile.

## 2 Methods

### 2.1 Subjects and study design

The present study was a single-center, randomized, open-label, placebo-controlled trial conducted on healthy Chinese subjects to evaluate the effect of the ET-26 blood concentration on the QT interval using the C-QTc model. This study aimed to investigate the pharmacokinetic characteristics and safety profiles of ET-26 and its primary metabolite etomidate acid (ETA) (Yu et al., 2020). The study strictly adhered to the established protocol and complied with relevant laws and regulations governing drug clinical trial quality management, as well as the guidelines set forth by the ethics committee. It has been registered under the identification number CTR20233230 at the dedicated platform for registering clinical trials (http://www.chinadrugtrials.org.cn). Informed consent was obtained from all subjects before they participated in the clinical trial.

The study enrolled healthy male and female subjects. The exclusion criteria included high-risk factors for torsades de pointes such as hypokalemia, hypomagnesemia, bradycardia, heart failure, and recent myocardial infarction. Subjects with abnormal 12-lead ECG results and a baseline QT interval corrected by Fridericia formula (QTcF) ≥ 450 ms, PR interval ≥200 ms, and QRS wave duration ≥120 ms were also excluded. Additionally, subjects with potentially difficult airways (Mallampati grade III–IV) or those who had taken medications within 30 days prior to the trial were excluded.

In this trial, eighteen healthy subjects were assigned to two dosage groups: the low-dose group (0.8 mg/kg, the recommended Phase III dose) and the high-dose group (2.8 mg/kg, the highest well-tolerated dose in Phase I), with nine subjects in each group. Additionally, a placebo control group was established at a 2:1 ratio from both dosage groups, comprising three subjects from each group, totaling six subjects in the placebo group. Throughout the trial, all participants received intravenous injections of either 0.8 mg/kg or 2.8 mg/kg ET-26-HCl or placebo (physiological saline) in ascending order. Blood samples and ECG data were collected.

### 2.2 Pharmacokinetics and ECG data

The subjects were instructed to fast for at least 10 h before receiving the experimental drug. The low-dose group received the injection over a duration of 60 ± 5 s, while the high-dose group received it at a rate of 1.2 mg/kg/min for approximately 140 ± 10 s. Subjects strictly adhered to fasting conditions for 4 h following drug administration, with no water intake permitted for 2 h before or after drug administration. A standardized diet was provided, starting 4 h after drug administration.

In the low-dose group, venous blood samples were collected before dosing (within 60 min before administration) and at various time intervals after administration, including 0.0167, 0.0333, 0.05, 0.0833, 0.117, 0.167, 0.25, 0.417, 0.667, 1, 1.5, 2, 3, 4, 6, 8, and 24 h. An additional venous blood sample was collected immediately after dosing in the high-dose group. The collected blood samples were centrifuged for 10 minutes (1,700 g, 4 °C), and the plasma was subsequently stored at -70 °C for further analysis. Dynamic ECG machines were utilized in this study to acquire consistent ECG data parameters, such as heart rate, PR interval, QRS duration, QT interval, QTcF, and QTcB. The ECG data were collected simultaneously with the blood samples at various time intervals. Furthermore, baseline ECG data were obtained 2 days prior to dosing. Modified observer’s assessment of alertness/sedation (MOAA/S) score evaluations were performed every 120 ± 30 s after administration until three consecutive scores ≥5 were achieved. Eyelash reflexes were assessed concurrently with the MOAA/S scoring. Moreover, continuous ECG monitoring was conducted using a cardiac monitor from the start of dosing until three consecutive MOAA/S scores of ≥5 were achieved. One set of 12-lead ECG was obtained at 2, 4, 8, and 24 h post-administration and on the day before dosing. The placebo group was not subjected to MOAA/S scoring, eyelash reflex evaluation, or continuous ECG monitoring.

A validated liquid chromatography-tandem mass spectrometry (LC-MS/MS) method was employed to determine the concentrations of ET-26 and ETA in plasma samples collected at various time points. The analysis was performed using an AB SCIEX API 4000 mass spectrometer equipped with a C18 column (Phenomenex Luna, 2.0 mm ID × 50 mm). The mobile phase consisted of (A) a salt solution prepared by mixing ultrapure water (1:1,000, v/v) with (B) methanol. ET-26 and ETA were analyzed using positive ion mode electrospray ionization with multiple reaction monitoring. The standard curve ranges for ET-26 were 10.0–4,000 ng/mL, and for ETA, they were 2.0–800 ng/mL. For ET-26, intra-day accuracy ranged from -6.37% to 3.30%, while for ETA, the range was -3.48% to -0.91%. The maximum precision for ET-26 and ETA was 5.08% and 6.73%, respectively. The pharmacokinetic parameters of ET-26/ETA, including the peak concentration (*C*_max_), time to reach peak concentration (*T*_max_), area under the curve from 0 to the last measurable concentration (*AUC*_*0-t*_) and from 0 to infinity (*AUC*_*0-∞*_), elimination half-life (*t*_*1/2*_), apparent clearance rate (*CL*), apparent volume of distribution during terminal phase (*V*_*z*_), and mean residence time (*MRT*), were estimated using Phoenix WinNonlin software version 8.1 or above with a non-compartmental model.

### 2.3 Safety

The safety assessment in this trial encompassed various aspects, including the frequency and incidence rate of AEs. It also involves physical examinations, pre- and post-medication vital sign assessments, MOAA/S score evaluations, eyelash reflex examinations, 12-lead ECG analysis, and laboratory tests, such as complete blood count, blood biochemistry analysis, coagulation function test, and urinalysis. These safety evaluation indicators are widely acknowledged as reliable and accurate.

### 2.4 C-QTc model

This experiment utilized a linear mixed-effects C-QTc model to investigate the relationship between ΔQTcF and blood concentrations of ET-26 and ETA. ΔQTcF refers to QTcF corrected by baseline, which is calculated as the average of QTcF values at different time points minus their respective baseline values. The model considered post-dose ΔQTcF as the dependent variable and examined the effects of medication (experimental drug, placebo), blood concentrations of ET-26/ETA, sampling time points, and centered the QTcF baseline as independent variables. The centered QTcF baseline for each participant was calculated by subtracting the individual baseline QTcF values from the mean baseline QTcF values of all participants within their respective medication groups. Additionally, random effects were incorporated in conjunction with intercepts to account for variations in the blood concentration levels of ET-26/ETA. Detailed information can be found in Formula 1.

$$∆QTcF_{ijk}=\left(\theta_{0}+\eta_{0,i}\right)+\theta_{1}TRT_{j}+\left(\theta_{2}+\eta_{2,i}\right)C_{ijk}+\theta_{3}TIME_{k}+\theta_{4}\left({QTcF}_{ij*}-\bar{{QTcF}_{j*}}\right)$$ (1)

ΔQTcF_*ijk*_ represents the change in QTcF for subject *i* under treatment *j* at time point *k*. *θ*_0_ and *η*_0,*i*_ are the fixed and random effects of the intercept term respectively. TRT*j* indicates treatment (1 for experimental drug, 2 for placebo), and *θ*_1_ is the fixed effect of treatment. C_*ijk*_ represents the blood concentration of ET-26/ETA at time point *k* for subject *i* under treatment *j*, while *θ*_2_ and *η*_*2,i*_ represent the fixed and random effects of blood concentration of ET-26/ETA respectively. *TIME* refers to the time points where PK blood tests and QTcF measurements were conducted; *θ*_3_ is the fixed effect of measurement time. *θ*_4_ is the fixed effect of baseline, where QTcF_ij_ represents the mean value of QTcF at baseline for subject *i* under treatment *j*. $\bar{{QTcF}_{j*}}$ is the mean value across all baseline values.

The C-QTc model analysis was employed using R software (version 4.3.2) and SAS software (version 9.4) to simulate the correlation between ∆QTcF and blood drug concentration. Utilizing the C-QTc model, we assessed the upper limit of the 90% two-sided confidence interval (CI) for QTcF interval corrected by baseline and placebo (ΔΔQTcF) values corresponding to the geometric mean of ET-26 and ETA *C*_max_ at clinically relevant doses.

## 3 Results

### 3.1 Population

The study enrolled 18 healthy Chinese participants, with an equal distribution of six individuals each in the low-dose, high-dose, and placebo groups. Demographic details are presented in Table 1. Overall, there were 14 males and four females, with an average age of 31.2 years. The participants had a mean height of 170.2 cm, a mean weight of 67.6 kg, and a mean body mass index (BMI) of 23.3 kg/m^2^. No statistically significant differences in these characteristics were observed between groups when analyzed using a t-test.

**TABLE 1:** The baseline characters of subjects.

Parameters (unit)	Total	Low-dose	High-dose	Placebo
Sex (male/female)	18 (14/6)	6 (5/1)	6 (6/0)	6 (3/3)
Age (years)	31.2 ± 5.7	33.2 ± 5.5	31.0 ± 3.6	29.3 ± 7.0
Height (cm)	170.2 ± 7.0	171.2 ± 6.5	171.5 ± 5.2	167.8 ± 8.3
Weight (kg)	67.6 ± 9.5	67.7 ± 10.6	69.7 ± 5.0	65.4 ± 11.1
BMI (kg/m^2^)	23.3 ± 2.5	23.1 ± 2.8	23.8 ± 2.2	23.1 ± 2.4

### 3.2 Pharmacokinetic parameters

**FIGURE 1:**
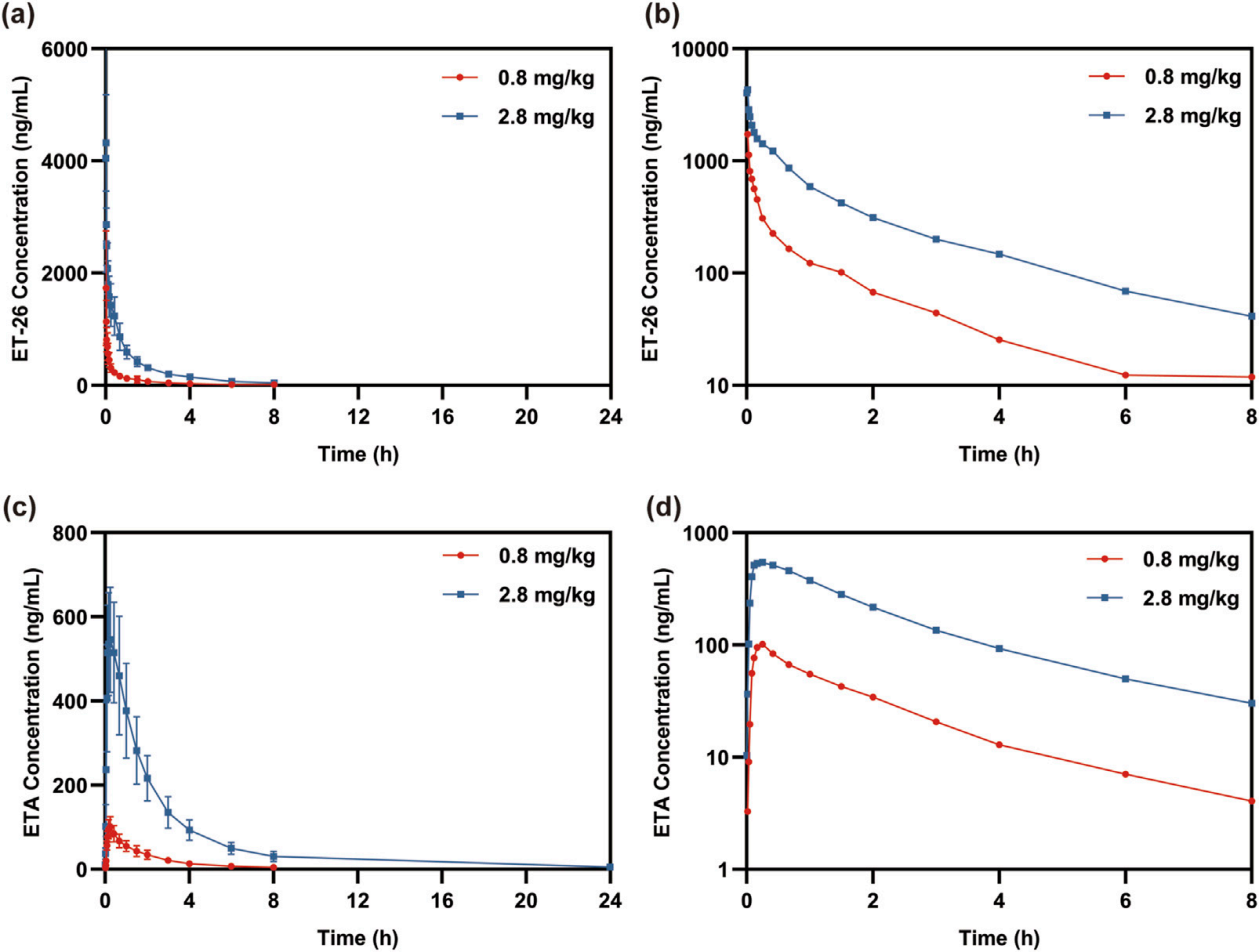
The time curve of the mean concentration of **(a)**, **(b)** methoxyetomidate (ET-26) and **(c)**, **(d)** etomidate acid (ETA).

All participants in the low and high-dose groups completed the experiments. Figure 1 illustrates the drug–time curve of ET-26 and its metabolite ETA, and Table 2 shows the PK parameters. With increasing dosage, the *C*_max_ of ET-26 in the high-dose group showed a roughly 2.7-fold increase, and the *AUC* demonstrated an approximately 4.3-fold rise. Regarding ETA, the *C*_max_ in the high-dose group was 5.5 times higher than that in the low-dose group, with a corresponding *AUC* elevation of approximately 7.7-fold. Both ET-26 and ETA showed an approximately 1.5-fold increase in the *MRT* in the high-dose group.

**TABLE 2:** The pharmacokinetic (PK) parameters of methoxetamine (ET-26) and its metabolite etomidate acid (ETA).

	ET-26		ETA	
Parameters (units)	Low-dose	High-dose	Low-dose	High-dose
T_max_ (min)	2.02 (2.00–2.13)	3.33 (2.33–3.40)	16.00 (11.02–16.02)	17.33 (7.33–27.33)
C_max_ (ng/mL)	1730 ± 1,020	4,720 ± 1,040	104 ± 24.8	572 ± 115
AUC_0-t_ (h·ng/mL)	613 ± 203	2,620 ± 491	191 ± 52.2	1,480 ± 488
AUC_0-∞_ (h·ng/mL)	649 ± 200	2,750 ± 527	204 ± 0.3	1,520 ± 488
t_1/2_ (h)	1.48 ± 0.345	2.01 ± 0.240	2.14 ± 0.474	4.10 ± 1.73
MRT_0-t_ (h)	1.02 ± 0.23	1.57 ± 0.19	2.04 ± 0.17	3.15 ± 0.89
MRT_0-∞_ (h)	1.39 ± 0.26	1.99 ± 0.35	2.61 ± 0.43	3.67 ± 0.97
CL (L/h)	88.5 ± 29.5	73.2 ± 13.7		
Vd (L)	181 ± 36.9	211 ± 40.9		

### 3.3 Model independent checks of assumptions

A linear mixed-effects C-QTc model was employed in this study to investigate the correlation between ΔQTcF and the ET-26 and ETA concentration. The analysis encompassed the baseline period and post-administration ECG data, along with the corresponding concentration data of the placebo, low-dose, and high-dose groups. When summarizing QTcF and ΔQTcF data, it was observed that expect for one subject in the placebo group who exhibited a ΔQTcF >30 ms and ≤60 ms at 1, 2, and 3 h post-administration, all other subjects had QTcF values ≤450 ms at various time points, and ΔQTcF values ≤30 ms, as described in Table 3. The establishment of a C-QTc model was based on the evaluation of key assumptions regarding the C-QTc relationship, as recommended in the scientific white paper on modeling C-QTc (Garnett et al., 2017; Garnett et al., 2018), and the assumptions of this experimental model were evaluated through graphics.

**TABLE 3:** The descriptive analysis results for QTcF and ΔQTcF.

Parameters	Low-dose	High-dose	Placebo
Baseline			
QTcF, ms			
≤450	6 (100%)	6 (100%)	6 (100%)
>450 and ≤ 480	0 (0)	0 (0)	0 (0)
>450 and ≤ 480	0 (0)	0 (0)	0 (0)
>500	0 (0)	0 (0)	0 (0)
0 min			
QTcF, ms			
≤450		6 (100%)	3 (100%)
>450 and ≤ 480		0 (0)	0 (0)
>450 and ≤ 480		0 (0)	0 (0)
>500		0 (0)	0 (0)
ΔQTcF, ms			
≤30		6 (100%)	3 (100%)
>30 and ≤ 60		0 (0)	0 (0)
>60		0 (0)	0 (0)
1 min			
QTcF, ms			
≤450	6 (100%)	6 (100%)	6 (100%)
>450 and ≤ 480	0 (0)	0 (0)	0 (0)
>450 and ≤ 480	0 (0)	0 (0)	0 (0)
>500	0 (0)	0 (0)	0 (0)
ΔQTcF, ms			
≤30	6 (100%)	6 (100%)	6 (100%)
>30 and ≤ 60	0 (0)	0 (0)	0 (0)
>60	0 (0)	0 (0)	0 (0)
2 min			
QTcF, ms			
≤450	6 (100%)	6 (100%)	6 (100%)
>450 and ≤ 480	0 (0)	0 (0)	0 (0)
>450 and ≤ 480	0 (0)	0 (0)	0 (0)
>500	0 (0)	0 (0)	0 (0)
ΔQTcF, ms			
≤30	6 (100%)	6 (100%)	6 (100%)
>30 and ≤ 60	0 (0)	0 (0)	0 (0)
>60	0 (0)	0 (0)	0 (0)
3 min			
QTcF, ms			
≤450	6 (100%)	6 (100%)	6 (100%)
>450 and ≤ 480	0 (0)	0 (0)	0 (0)
>450 and ≤ 480	0 (0)	0 (0)	0 (0)
>500	0 (0)	0 (0)	0 (0)
ΔQTcF, ms			
≤30	6 (100%)	6 (100%)	6 (100%)
>30 and ≤ 60	0 (0)	0 (0)	0 (0)
>60	0 (0)	0 (0)	0 (0)
5min			
QTcF, ms			
≤450	6 (100%)	6 (100%)	6 (100%)
>450 and ≤ 480	0 (0)	0 (0)	0 (0)
>450 and ≤ 480	0 (0)	0 (0)	0 (0)
>500	0 (0)	0 (0)	0 (0)
ΔQTcF, ms			
≤30	6 (100%)	6 (100%)	6 (100%)
>30 and ≤ 60	0 (0)	0 (0)	0 (0)
>60	0 (0)	0 (0)	0 (0)
7 min			
QTcF, ms			
≤450	6 (100%)	6 (100%)	6 (100%)
>450 and ≤ 480	0 (0)	0 (0)	0 (0)
>450 and ≤ 480	0 (0)	0 (0)	0 (0)
>500	0 (0)	0 (0)	0 (0)
ΔQTcF, ms			
≤30	6 (100%)	6 (100%)	6 (100%)
>30 and ≤ 60	0 (0)	0 (0)	0 (0)
>60	0 (0)	0 (0)	0 (0)
10 min			
QTcF, ms			
≤450	6 (100%)	6 (100%)	6 (100%)
>450 and ≤ 480	0 (0)	0 (0)	0 (0)
>450 and ≤ 480	0 (0)	0 (0)	0 (0)
>500	0 (0)	0 (0)	0 (0)
ΔQTcF, ms			
≤30	6 (100%)	6 (100%)	6 (100%)
>30 and ≤ 60	0 (0)	0 (0)	0 (0)
>60	0 (0)	0 (0)	0 (0)
15 min			
QTcF, ms			
≤450	6 (100%)	6 (100%)	6 (100%)
>450 and ≤ 480	0 (0)	0 (0)	0 (0)
>450 and ≤ 480	0 (0)	0 (0)	0 (0)
>500	0 (0)	0 (0)	0 (0)
ΔQTcF, ms			
≤30	6 (100%)	6 (100%)	6 (100%)
>30 and ≤ 60	0 (0)	0 (0)	0 (0)
>60	0 (0)	0 (0)	0 (0)
25 min			
QTcF, ms			
≤450	6 (100%)	6 (100%)	6 (100%)
>450 and ≤ 480	0 (0)	0 (0)	0 (0)
>450 and ≤ 480	0 (0)	0 (0)	0 (0)
>500	0 (0)	0 (0)	0 (0)
ΔQTcF, ms			
≤30	6 (100%)	6 (100%)	6 (100%)
>30 and ≤ 60	0 (0)	0 (0)	0 (0)
>60	0 (0)	0 (0)	0 (0)
40 min			
QTcF, ms			
≤450	6 (100%)	6 (100%)	6 (100%)
>450 and ≤ 480	0 (0)	0 (0)	0 (0)
>450 and ≤ 480	0 (0)	0 (0)	0 (0)
>500	0 (0)	0 (0)	0 (0)
ΔQTcF, ms			
≤30	6 (100%)	6 (100%)	6 (100%)
>30 and ≤ 60	0 (0)	0 (0)	0 (0)
>60	0 (0)	0 (0)	0 (0)
1 h			
QTcF, ms			
≤450	6 (100%)	6 (100%)	6 (100%)
>450 and ≤ 480	0 (0)	0 (0)	0 (0)
>450 and ≤ 480	0 (0)	0 (0)	0 (0)
>500	0 (0)	0 (0)	0 (0)
ΔQTcF, ms			
≤30	6 (100%)	6 (100%)	5 (83.3%)
>30 and ≤ 60	0 (0)	0 (0)	1 (16.7%)
>60	0 (0)	0 (0)	0 (0)
1.5 h			
QTcF, ms			
≤450	6 (100%)	6 (100%)	6 (100%)
>450 and ≤ 480	0 (0)	0 (0)	0 (0)
>450 and ≤ 480	0 (0)	0 (0)	0 (0)
>500	0 (0)	0 (0)	0 (0)
ΔQTcF, ms			
≤30	6 (100%)	6 (100%)	6 (100%)
>30 and ≤ 60	0 (0)	0 (0)	0 (0)
>60	0 (0)	0 (0)	0 (0)
2 h			
QTcF, ms			
≤450	6 (100%)	6 (100%)	6 (100%)
>450 and ≤ 480	0 (0)	0 (0)	0 (0)
>450 and ≤ 480	0 (0)	0 (0)	0 (0)
>500	0 (0)	0 (0)	0 (0)
ΔQTcF, ms			
≤30	6 (100%)	6 (100%)	6 (100%)
>30 and ≤ 60	0 (0)	0 (0)	0 (0)
>60	0 (0)	0 (0)	0 (0)
3 h			
QTcF, ms			
≤450	6 (100%)	6 (100%)	6 (100%)
>450 and ≤ 480	0 (0)	0 (0)	0 (0)
>450 and ≤ 480	0 (0)	0 (0)	0 (0)
>500	0 (0)	0 (0)	0 (0)
ΔQTcF, ms			
≤30	6 (100%)	6 (100%)	6 (100%)
>30 and ≤ 60	0 (0)	0 (0)	0 (0)
>60	0 (0)	0 (0)	0 (0)
4 h			
QTcF, ms			
≤450	6 (100%)	6 (100%)	6 (100%)
>450 and ≤ 480	0 (0)	0 (0)	0 (0)
>450 and ≤ 480	0 (0)	0 (0)	0 (0)
>500	0 (0)	0 (0)	0 (0)
ΔQTcF, ms			
≤30	6 (100%)	6 (100%)	6 (100%)
>30 and ≤ 60	0 (0)	0 (0)	0 (0)
>60	0 (0)	0 (0)	0 (0)
6 h			
QTcF, ms			
≤450	6 (100%)	6 (100%)	6 (100%)
>450 and ≤ 480	0 (0)	0 (0)	0 (0)
>450 and ≤ 480	0 (0)	0 (0)	0 (0)
>500	0 (0)	0 (0)	0 (0)
ΔQTcF, ms			
≤30	6 (100%)	6 (100%)	6 (100%)
>30 and ≤ 60	0 (0)	0 (0)	0 (0)
>60	0 (0)	0 (0)	0 (0)
8 h			
QTcF, ms			
≤450	6 (100%)	6 (100%)	6 (100%)
>450 and ≤ 480	0 (0)	0 (0)	0 (0)
>450 and ≤ 480	0 (0)	0 (0)	0 (0)
>500	0 (0)	0 (0)	0 (0)
ΔQTcF, ms			
≤30	6 (100%)	6 (100%)	6 (100%)
>30 and ≤ 60	0 (0)	0 (0)	0 (0)
>60	0 (0)	0 (0)	0 (0)
24 h			
QTcF, ms			
≤450	6 (100%)	6 (100%)	6 (100%)
>450 and ≤ 480	0 (0)	0 (0)	0 (0)
>450 and ≤ 480	0 (0)	0 (0)	0 (0)
>500	0 (0)	0 (0)	0 (0)
ΔQTcF, ms			
≤30	6 (100%)	6 (100%)	6 (100%)
>30 and ≤ 60	0 (0)	0 (0)	0 (0)
>60	0 (0)	0 (0)	0 (0)

#### 3.3.1 Assumption 1: the experimental drug did not affect heart rate

**FIGURE 2:**
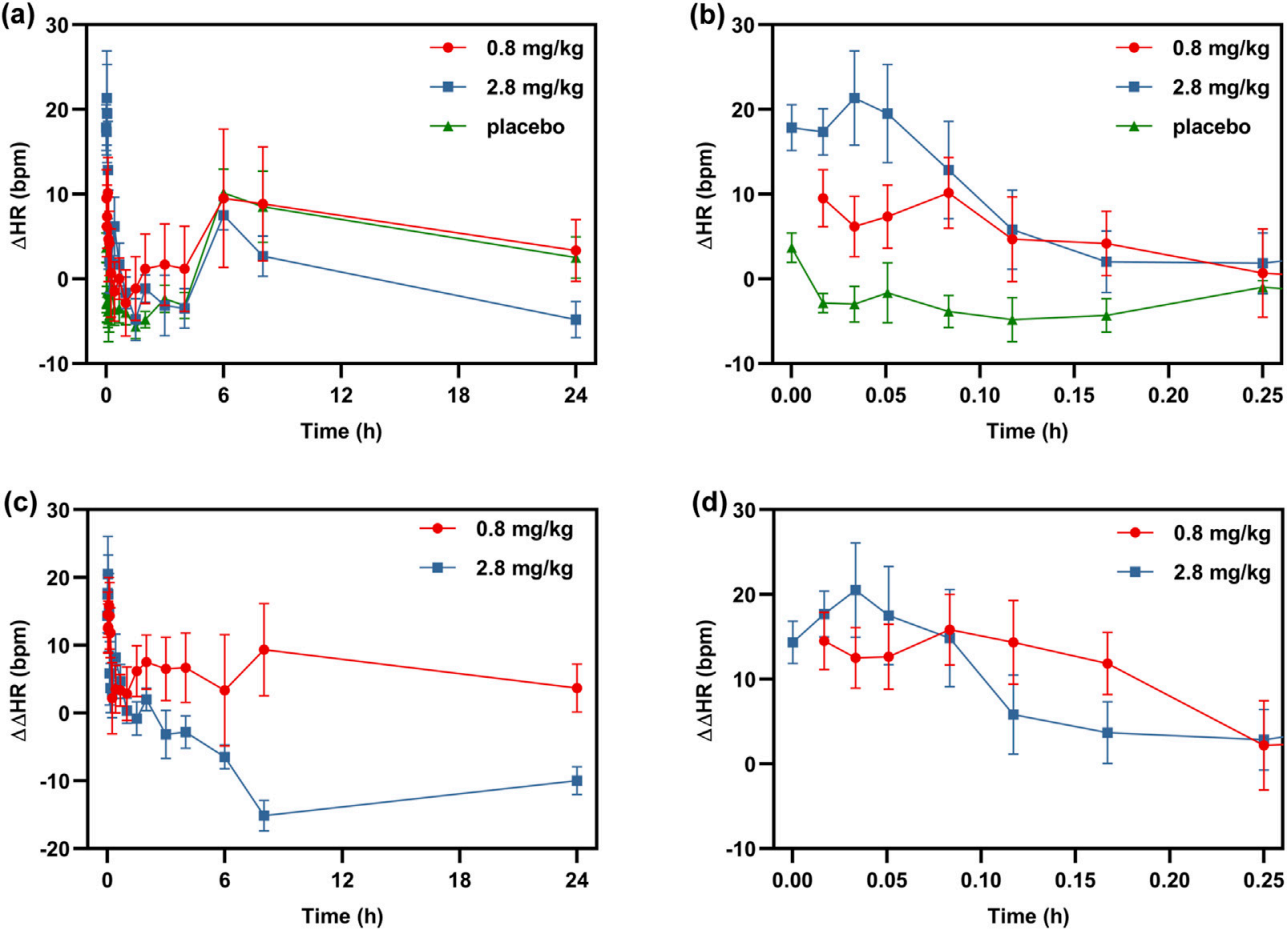
The heart rate profiles of each group at various time points. **(a)**, **(b)** baseline corrected, **(c)**, **(d)** baseline and placebo corrected.

The heart rate profiles of the low-dose, high-dose, and placebo groups at various time points are depicted in Figures 2a, b after baseline correction. Figures 2c, d illustrate the corrected heart rate profiles of the baseline and placebo groups, respectively. These findings suggest that the impact on heart rate increases proportionally with dosage within 3 min of administration. Between 5 and 10 mins, both the low and high-dose groups exhibited similar yet slightly elevated changes in heart rate compared to the placebo group. After 15 min post-administration, the effects on participants’ heart rates remained consistent across all three groups.

#### 3.3.2 Assumption 2: the QTc interval remains unaffected by heart rate

**FIGURE 3:**
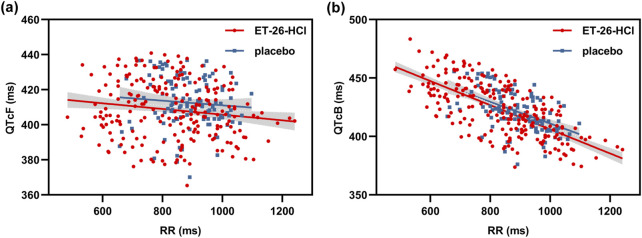
The scatter plots of QT interval corrected by **(a)** the fridericia formula (qtcf) and **(b)** Bazett formula (QTcB) against RR for the ET-26-HCl and placebo.

The QT intervals of the two dosage and placebo groups were corrected using either the Fridericia or Bazett formula. Scatter plots illustrating the relationship between QTcF/QTcB and RR for ET-26 and the placebo are shown in Figure 3. Compared to QTcB, the regression line slope for the QTcF correction approaches zero more closely. The distribution of QTcF corresponding to heart rate appeared random on both sides of the linear regression, indicating that heart rate did not influence on QTcF.

#### 3.3.3 Assumption 3: there is no temporal delay effect observed between the alteration in drug concentration and ΔΔQTcF

**FIGURE 4:**
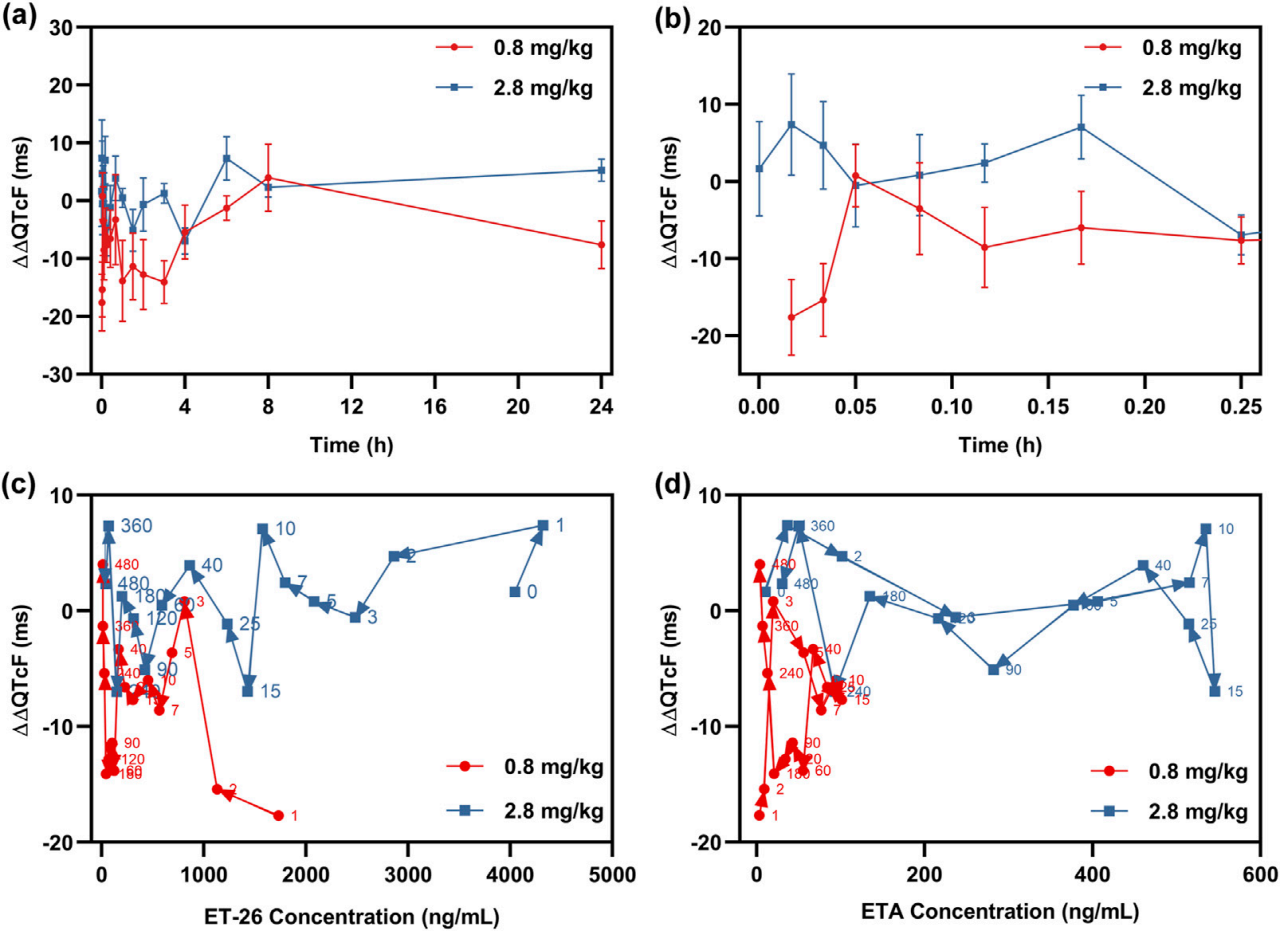
**(a)**, **(b)** the line graphs of qtcf intervals corrected for baseline and placebo (δδqtcf) versus each sampling point; the correlation profiles line of δδqtcf versus the concentration of **(c)** et-26 and **(d)** ETA.

The QTcF interval versus time curves for each dose group, adjusted for baseline and placebo, are presented in Figure 4. Following the completion of administration, the subjects exhibited a state of nap rest with decreased heart rate and a prolonged QT interval lasting for 6–8 h, as indicated by their living status at the clinical center. The results depicted in Figures 1, 4 indicate that within 15 min after the administration ended, the low-dose group exhibited the highest ΔΔQTcF value at 3 min. Additionally, ET-26 and ETA achieved peak blood concentrations at 1 and 15 min, respectively. Conversely, in the high-dose group, the maximum ΔΔQTcF value was observed at 7 min, with ET-26 and ETA reaching their respective peak blood drug concentrations at 1 and 15 min, as observed in the low-dose group. These findings suggest a correlation between ΔΔQTcF values and changes in blood drug concentrations without any evident delayed effect.

#### 3.3.4 Assumption 4: the relationship between ΔQTcF and drug concentration is linear

**FIGURE 5:**
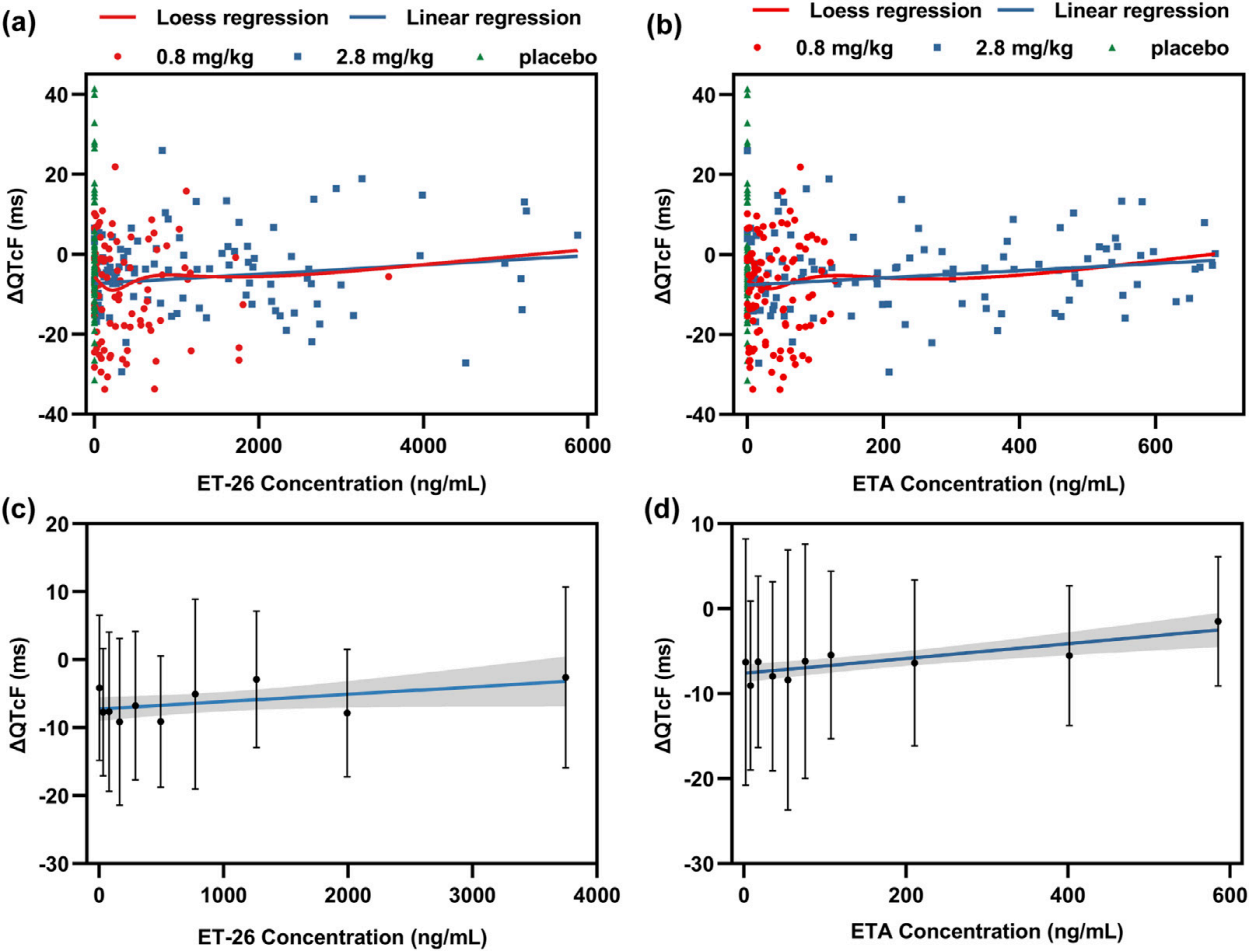
The scatter plot of **(a)** ET-26 and **(b)** ETA concentrations and QTcF corrected by baseline (ΔQTcF) in all experiment groups. The tenth-order dispersed point plot of the correlation between ΔQTcF and **(c)** ET-26 and **(d)** ETA. The red line represents the Loess regression model, while the blue line represents the linear regression model. The shaded area corresponds to the 90% confidence interval of the regression line.

The scatter plots in Figure 5 illustrate the overlap between the locally estimated scatterplot smoothing (LOESS) regression and linear regression for the baseline-corrected QTcF interval and blood concentrations of ET-26 and ETA in each dosage group. By dividing the overall range of all data concentrations into 10 equal intervals, a positive correlation was observed between ΔQTcF and the drug concentrations of ET-26 and ETA within each interval.

### 3.4 C-QTc modelling

The four hypotheses mentioned above were not contradicted by any apparent evidence found in this experiment, indicating the continued validity of the assumptions. A linear mixed-effects model was employed to establish a C-QTc model, with the ET-26/ETA blood drug concentration, sampling time points, and centralized QTcF baseline as the independent variables and ΔQTcF as the dependent variable. The established model parameters are listed in Table 4.

**TABLE 4:** The estimation of parameters in linear mixed models of ET-26 and ETA.

Model parameters	ET-26		ETA	
	Estimated value	90% CI	Estimated value	90% CI
Intercept, ms	−3.761	[−8.527; 1.005]	−3.946	[−5.774; −2.118]
Administration, ms	−3.793	[−9.670; 2.085]	−5.110	[−7.511; −2.709]
Sampling time point				
0 h	−3.793	[−9.670; 2.085]	−5.110	[−7.511; −2.709]
1 min	−3.789	[−9.667; 2.088]	−5.106	[−7.506; −2.705]
2 min	−3.786	[−9.664; 2.091]	−5.102	[−7.502; −2.702]
3 min	−3.783	[−9.660; 2.094]	−5.098	[−7.497; −2.698]
5 min	−3.777	[−9.654; 2.101]	−5.089	[−7.488; −2.690]
7 min	−3.770	[−9.647; 2.107]	−5.081	[−7.479; −2.683]
10 min	−3.760	[−9.636; 2.116]	−5.068	[−7.465; −2.671]
15 min	−3.744	[−9.620; 2.132]	−5.047	[−7.443; −2.652]
25 min	−3.711	[−9.586; 2.164]	−5.005	[−7.398; −2.613]
40 min	−3.662	[−9.535; 2.212]	−4.943	[−7.332; −2.554]
1 h	−3.596	[−9.468; 2.276]	−4.859	[−7.244; −2.474]
1.5 h	−3.498	[−9.369; 2.373]	−4.734	[−7.117; −2.351]
2 h	−3.399	[−9.270; 2.471]	−4.608	[−6.992; −2.224]
3 h	−3.203	[−9.075; 2.670]	−4.357	[-6.755; −1.960]
4 h	−3.006	[−8.889; 2.872]	−4.106	[−6.531; −1.682]
6 h	−2.612	[−8.511; 3.287]	−3.604	[−6.124; −1.085]
8 h	−2.219	[−8.154; 3.716]	−3.102	[-5.766; −0.439]
24 h	0.929	[−5.792; 7.650]	0.913	[−3.874; 5.700]
Blood concentration (slope), ng/mL	0.001	[−0.001; 0.003]	0.013	[−0.001; 0.027]
Baseline, ms	−0.202	[−0.393; −0.011]	−0.242	[−0.321; −0.163]
Estimated variance				
Intercept, ms^2^	39.911		0.000061	
Blood concentration, (ng/mL)^2^	0.000006		0.000061	
Residual, ms^2^	80.263		117.550	

The final models produced estimated slopes of 0.001 ng/mL (90% CI: −0.001–0.003 ng/mL) for ET-26 and 0.013 ng/mL (90% CI: −0.001–0.027 ng/mL) for ETA, indicating a limited correlation between the concentrations of ET-26 and ETA with ΔQTcF. Importantly, all parameters exhibited insignificant standard errors, implying that the estimates closely approximated their true values with minimal variability among the data points.

**FIGURE 6:**
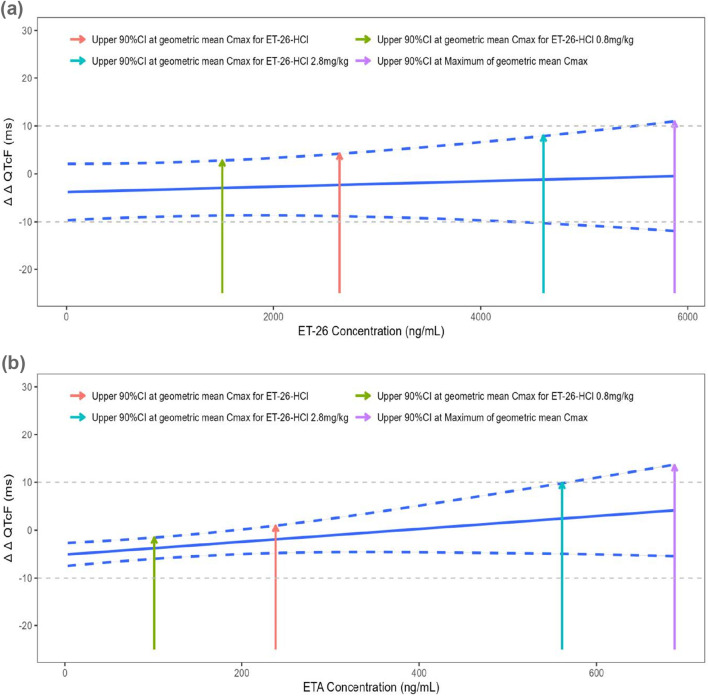
The correction plot of predicted ΔΔQTcF with the concentrations of **(a)** ET-26 and **(b)** ETA.

The mean ΔΔQTcF value and upper limit of the 90% CI for *C*_max_, predicted using a linear mixed-effects model, are presented in Figure 6; Table 5. The geometric mean (GM) of ET-26 *C*_max_ was 2,640 ng/mL, resulting in a mean ΔΔQTcF value of −2.305 ms with an upper limit of the 90% CI at 4.204 ms, which did not exceed the predefined threshold value of 10 ms. In contrast, ETA exhibited *C*_max_ levels at 238 ng/mL, corresponding to a mean ΔΔQTcF value of −1.913 ms with an upper limit below the predetermined threshold value set at 10 ms (0.936 ms). However, both ET-26 and ETA showed maximum *C*_max_ values that exceeded the predefined threshold values.

**TABLE 5:** The summary of the mean predicted QTcF interval corrected by baseline and placebo (ΔΔQTcF) and upper limit of the 90% confidence interval (CI) corresponding to the geometric mean (GM) of peak concentration (*C*_max_) for ET-26 and ETA.

Predicted ΔΔQTcF	Ls mean (upper limit of 90% CI)
ET-26	
Total GM^*^ C_max_ = 2,640 ng/mL	−2.305 (4.204) ms
0.8 mg/kg GM C_max_ = 1,510 ng/mL	−2.942 (2.788) ms
2.8 mg/kg GM C_max_ = 4,610 ng/mL	−1.192 (8.010) ms
Max C_max_ = 5,870 ng/mL	−0.478 (10.895) ms
ETA	
Total GM C_max_ = 238 ng/mL	−1.913 (0.936) ms
0.8 mg/kg GM C_max_ = 101 ng/mL	−3.754 (−1.543) ms
2.8 mg/kg GM C_max_ = 561 ng/mL	2.428 (9.880) ms
Max C_max_ = 688 ng/mL	4.134 (13.551) ms

### 3.5 Pharmacodynamics

**FIGURE 7:**
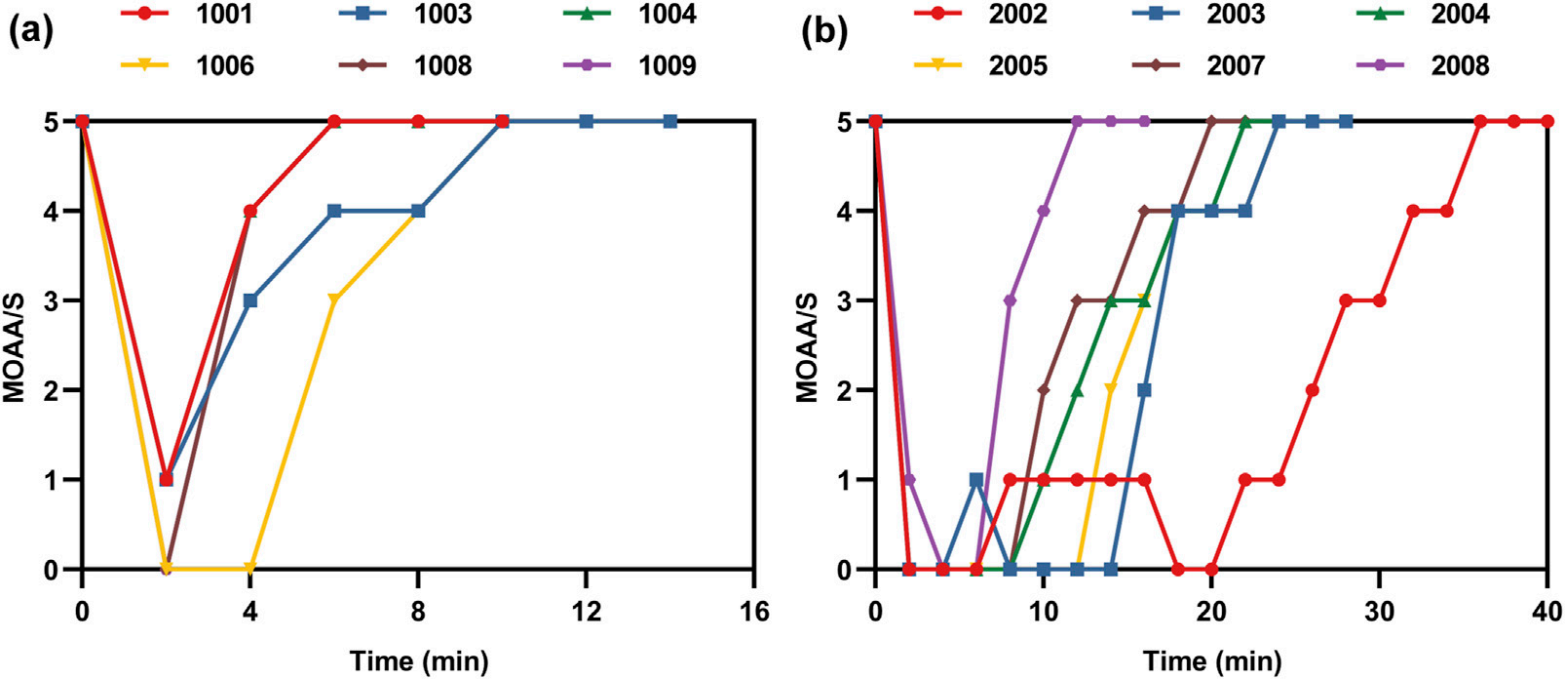
Modified observer’s assessment of alertness/sedation (MOAA/S) scores by subject, **(a)** low-dose group, **(b)** high-dose group.

The clinical efficacy of the experimental drug was evaluated using the MOAA/S score and eyelash reflex assessment. The changes in the MOAA/S scores for each subject are shown in Figure 7. In the low-dose group, the median duration of loss of consciousness (MOAA/S score ≤1) was 2.30 min, while it was observed to be 2.20 min in the high-dose group. Regarding the eyelash reflex, 83.3% of the subjects (5/6) consistently maintained their eyelash reflex throughout the experiment in the low-dose group. Conversely, all six subjects in the high-dose group experienced temporary disappearance and subsequent recovery of their eyelash reflex after drug administration, with the disappearance time ranging from 2 to 4 min and recovery time ranging from 11 to 22 min. ET-26 exhibited a rapid onset of action and quick recovery, while also demonstrating dose-dependent sedative and hypnotic effects.

### 3.6 Safety

During the experiment, 10 subjects in the ET-26-HCl group experienced 19 treatment-emergent AEs (TEAEs), all of which were deemed related to the drug. Details of the TEAEs are listed in Table 6. The overall incidence rate of TEAEs was 55.6% (10/18). In the low-dose group, four subjects experienced six instances of TEAEs, with a severity level distribution of 83.3% grade 1 (5/6) and 16.7% grade 2 (1/6). In the high-dose group, six subjects experienced a total of 13 instances of TEAEs, all at the grade 1 severity level. Myoclonus was the most frequently observed TEAE, which occurred in nine subjects (9/12, 75%). Most TEAEs did not necessitate interventions except for one patient who received non-pharmacological treatment.

**TABLE 6:** The summary of treatment-emergent adverse events (TEAE).

System-organ classes	Low-dose	High-dose
Neurological diseases		
Myoclonus, I	3 (50.0%)	6 (46.1%)
Dyskinesia, I	2 (33.3%)	
Musculoskeletal and connective tissue disorders		
muscle rigidity, I		2 (15.4%)
Respiratory, chest and mediastinal diseases		
Hiccup, I		2 (15.4%)
Increased secretion of the upper respiratory tract, II		1 (7.7%)
Blood and lymphatic system disorders		
Anemia, II	1 (16.7%)	
Systemic diseases and various reactions at the site of administration		
Injection site pain, I		1 (7.7%)
Inspections		
Hypokalemia, I		1 (7.7%)

## 4 Discussion

The study aimed to assess the effect of ET-26 on the QT interval using the C-QTc model and to evaluate its PK characteristics in healthy subjects. In this experiment, the mean *C*_max_ value for ET-26 was 2,640 ng/mL, which corresponded to a mean ΔΔQTcF value of −2.305 m with an upper limit of the 90% CI at 4.204 m, <10 m. The upper limits of the 90% CI for ΔΔQTcF corresponding to ET-26 and ETA at twice the *C*_max_ in phase IIb (4,624 and 272.4 ng/mL, respectively) were both <10 m. The increase in *C*_max_ values for ET-26 demonstrated a proportional relationship with dosage escalation, while the *AUC*_*0-t*_ and *AUC*_*0-∞*_ slightly exceeded the proportional dosage increase. Additionally, the individual variability observed in this study was consistent with that of previous phase I clinical trials conducted using similar dosages. Exposure to ET-26 appears to be minimally influenced by special populations, drug–drug interactions (DDI), and other factors, as suggested by additional clinical findings.

Upon entering the bloodstream, ET-26-HCl is converted into ET-26, which has a structure similar to that of etomidate and serves as an anesthetic and sedative. ET-26 offers respiratory and cardiovascular stability within a wider safety range while weakening the inhibitory effect on adrenal cortex function. In this experiment, ET-26-HCl demonstrated significant sedative properties with a median duration of loss of consciousness after administration being 2.30 min in the low-dose group and 2.20 min in the high-dose group. The subjects experienced an average eyelash reflex disappearance time of 2 min and a recovery time of 11 min in the high-dose group. These results support previous reports by highlighting the favorable anesthesia and sedative effects of ET-26 characterized by a rapid onset and short recovery time (Jiang et al., 2024).

A concentration-QT (C-QT) analysis is generally necessary for most systemically administered drugs, even in the absence of preclinical evidence of ventricular repolarization effects (Lester et al., 2019). This requirement is primarily driven by regulatory guidelines such as ICH E14, which emphasize the need for human QT assessment unless a compelling case for exemption is provided (ICH, 2022). Preclinical models, while valuable, may not fully predict human cardiac responses due to species-specific differences, metabolic variations, and limited exposure ranges (Kathiresan and Srivastava, 2012). Additionally, QT prolongation can be concentration-dependent, meaning that higher doses or specific patient populations may exhibit effects not observed in preclinical studies (Shah, 2002). However, exceptions exist for drugs with minimal systemic exposure (e.g., topical agents), extremely short half-lives, or well-characterized mechanisms that present no plausible risk (ICH, 2015). In such cases, a scientifically justified waiver may be possible. Nonetheless, given the potential clinical implications of QT prolongation, conducting a C-QT analysis remains a critical component of drug safety evaluation unless substantial evidence indicates it is unnecessary. For ET-26, as with most drugs, proactive C-QT assessment ensures patient safety, regulatory compliance, and market confidence.

Previous studies have indicated that etomidate administration does not result in a prolonged QT interval (Lischke et al., 1994; Ay et al., 2003; Erdil et al., 2009), and there is currently no available literature establishing a C-QTc model for etomidate or similar compounds (Niimi et al., 2022). The present study developed C-QTc models for ET-26 and ETA, enabling the prediction of both the mean value and 90% CI of ΔΔQTcF corresponding to the *C*_max_ during the administration of ET-26-HCl. The model’s goodness-of-fit was assessed through diagnostic plots, including scatter plots depicting ΔQTcF residuals against ET-26 and ETA concentrations, a scatter plot illustrating ΔQTcF residuals against QTcF baseline, a contour plot, a boxplot, and a QQ plot based on ΔQTcF residuals and sampling time. The analysis based on these diagnostic plots indicated that the residuals exhibited random distribution around zero with a normal pattern, suggesting an excellent regression fit for the model.

The experimental drug ET-26-HCl demonstrated a favorable safety profile. The most frequently observed TEAEs associated with the drug were myoclonus in 75.0% of cases (9/12), dyskinesia in 16.7% (2/12), muscle rigidity in 16.7% (2/12), hiccup in 16.7% (2/12), increased upper respiratory secretions in 8.3% (1/12), decreased blood potassium levels in 8.3% (1/12), and injection site pain in 8.3% (1/12). These findings suggest that the AEs observed during this trial were consistent with those reported in previous clinical studies, indicating a correlation with the mechanism of action of the investigational drug.

The present study had certain limitations. First, the small sample size may have resulted in false positive findings, underscoring the necessity for more real-world data to facilitate a meaningful analysis. Second, there were variations in the male-to-female ratios among subjects in different groups, which could have also contributed to false-positive results. Third, the participants had relatively stable living conditions at the research center; however, drug users in real-world settings may experience greater fluctuations after medication intake, potentially leading to changes in the QTc interval. Finally, this study lacked data from elderly patients to demonstrate clinical therapeutic concentrations and their association with age-related increases in cardiovascular risk factors and concurrent medication treatments that impact the QTc interval. Further research is required to analyze the impact of ET-26-HCl on the QTc interval.

## 5 Conclusion

In this study, the exposure levels (*C*_max_, *AUC*_*0-t*_, *AUC*_*0-∞*_) of the active ingredient ET-26 and its metabolite ETA exhibited a proportional increase with dosage following a single intravenous injection of ET-26-HCl in healthy subjects. The observed mean and upper limit of the 90% CI for ΔΔQTcF corresponding to the *C*_max_ of ET-26 and ETA did not exceed 10 ms. Based on the established C-QTc model, it is predicted that the mean and upper limit of the 90% CI for ΔΔQTcF corresponding to twice the *C*_max_ of ET-26 and ETA in phase II clinical trials at applied dosages will be <10 ms, indicating no observed risk of QT prolongation from the prototype drug. Considering the clinical application of ET-26-HCl, close monitoring for safety is required when patients use this medication because of potential drug interactions and considerations related to special populations.

## Data Availability

The original contributions presented in the study are included in the article/supplementary material, further inquiries can be directed to the corresponding authors.
